# Growth differentiation factor 15 (GDF-15) as an emerging biomarker for cardiovascular and kidney diseases, a therapeutic target in cancer and a potential biomarker for preeclampsia

**DOI:** 10.3389/fphar.2026.1779087

**Published:** 2026-03-13

**Authors:** Daniela A. Pereira, Carla S. Ceron, Ricardo C. Cavalli, Valeria C. Sandrim, Marcelo R. Luizon

**Affiliations:** 1 Department of Biophysics and Pharmacology, Institute of Biosciences, Universidade Estadual Paulista (UNESP), Botucatu, São Paulo, Brazil; 2 Department of Genetics, Ecology and Evolution, Institute of Biological Sciences, Federal University of Minas Gerais, Belo Horizonte, Minas Gerais, Brazil; 3 Department of Biological Sciences, Institute of Exact and Biological Sciences, Federal University of Ouro Preto, Ouro Preto, Minas Gerais, Brazil; 4 Department of Gynecology and Obstetrics, Ribeirao Preto Medical School, University of Sao Paulo, Ribeirao Preto, São Paulo, Brazil

**Keywords:** cardiovascular diseases, endothelial dysfunction, genetic polymorphisms, growth differentiation factor 15, kidney diseases, nitric oxide, preeclampsia, cancer

## 1 Introduction

Growth Differentiation Factor 15 (GDF-15) was initially identified as macrophage inhibitory cytokine-1 (Bootcov et al., 1997), and is a stress-responsive cytokine within the transforming growth factor-beta superfamily (Hromas et al., 1997; Yokoyama-Kobayashi et al., 1997; Paralkar et al., 1998; Bottner et al., 1999). GDF-15 plays a central role in appetite and metabolic regulation by influencing lipolysis, thermogenesis, and feeding behavior (Klein et al., 2022). Notably, GDF-15 levels increase following administration of metformin, the most prescribed antidiabetic drug worldwide, and serve as an important mediator of its effects (Coll et al., 2020).

Circulating GDF-15 levels typically range from 100 to 1,200 pg/mL, but they can significantly rise in several physiological and pathological conditions, including aging, cancer, cardiovascular disease (CVD), metabolic disorders, and pregnancy (Adela and Banerjee, 2015; Tsai et al., 2018). Elevated GDF-15 levels have been associated with several cancers (Mimeault and Batra, 2010; Chen et al., 2016; Assadi et al., 2020; Siddiqui et al., 2022) and investigated as a diagnostic and prognostic biomarker, particularly due to its roles in tumor progression and cachexia (Mimeault and Batra, 2010; Wang et al., 2021). It may also exert protective effects in several organs, such as the heart, liver, kidneys, and lungs following injury or inflammation (Emmerson et al., 2018). Importantly, GDF-15 is recognized as a biomarker of cardiac hypertrophy, heart failure, atherosclerosis, and endothelial dysfunction (Adela and Banerjee, 2015).

In this opinion article, we discuss the emerging significance of GDF-15 in vascular physiology, focusing on its implications and mechanisms underlying endothelial dysfunction in CVD and renal disease, as a therapeutic target in cancer, and a potential biomarker in preeclampsia.

## 2 GDF-15 as an emerging biomarker for CVD and kidney disease

Although GDF-15 is minimally expressed in cardiovascular tissues under normal conditions, it is increasingly recognized as a potential biomarker and therapeutic target in CVD due to its cardioprotective effects (Kempf et al., 2006; Xu et al., 2006; Wollert et al., 2017). Its expression is upregulated in cardiomyocytes and cardiac tissue following injury, heart failure, ischemia, and atherosclerosis, and it is also induced in vascular smooth muscle cells, endothelial cells, and adipocytes under oxidative stress, vascular damage, and inflammation (Secchiero et al., 2007; Bermudez et al., 2008; Ding et al., 2009; Adela and Banerjee, 2015). However, current evidence suggests that these associations may be compensatory rather than causative (Wang et al., 2021).

Serum GDF-15 levels were found to be increased in type 2 diabetic rats induced with streptozotocin and associated with cardiac aging and inflammation (Almohaimeed et al., 2025). Treatment with human recombinant GDF-15 improved diastolic dysfunction and reduced cardiac inflammation in diabetic type 2 mice that were induced with high-fat diet supplementation and streptozotocin injection, suggesting that GDF-15 may be cardioprotective for both obesity and diabetic cardiomyopathy (Chan et al., 2024). This same treatment also protected mice from renal tubular and interstitial damage in experimental streptozotocin type 1 and db/db type 2 diabetes, but not from glomerular damage (Mazagova et al., 2013). Moreover, tubular and interstitial damage in both models of diabetes was increased in GDF15 knockout mice (Mazagova et al., 2013).

GDF15 knockout mice were submitted to kidney ischemia-reperfusion injury to examine its role in kidney clinical transplantation, and these mice showed exacerbated acute tubular injury and inflammation. Accordingly, the incidence of biopsy-proven acute rejection of kidney clinical transplantation was associated with low circulating GDF-15 levels (Liu et al., 2020). Moreover, serum GDF-15 levels were rapidly increased after mouse models of unilateral ischemia-reperfusion injury and unilateral ureteral obstruction injury, with a posterior reduction. Hypoxia- and oxidative stress-mediated proximal tubular cell injury were associated with an increased *GDF15* expression. Increased serum GDF-15 levels were suggested to serve as an early indicator of acute kidney injury specifically related to proximal tubular cell injury (Sawasawa et al., 2024).

A protective role of GDF-15 in atherosclerotic lesions was suggested in a study in which smaller atherosclerotic lesions at the aortic sinus and abdominal aorta were observed in an ApoE^−/−^ mouse model of atherosclerosis that overexpresses *GDF15* in the macrophages in comparison with ApoE^−/−^ mice (Johnen et al., 2012). However, male GDF15−/−LDLr−/−atherosclerotic mice exhibited an obesogenic phenotype but reduced atherosclerotic lesion development when compared with littermate controls. The development of atherosclerosis was not affected by GDF-15 deficiency in female mice (Davezac et al., 2025).

GDF15 expression is upregulated in vessel walls of atherosclerotic mice. Its deletion in apoE−/− mice reduced atherosclerotic lesion development, suggesting a regulatory role in IL-6–dependent inflammation, vascular injury, and apoptotic cell death during lesion progression (Bonaterra et al., 2012). In a mouse model of advanced atherosclerosis, GDF-15 presented a protective role in macrophage accumulation and features of atherosclerotic plaque destabilization (Preusch et al., 2013). However, global *GDF15* knockout did not affect atherosclerosis development in either sex, despite increased serum GDF-15 levels in ApoE^−/−^ mice; *GDF15* deletion or recombinant treatment modulated lipid deposition and macrophage polarization in foam cells (Liu et al., 2020).

A protective role of GDF-15 against pressure overload-induced cardiac hypertrophy was observed in mice overexpressing GDF15 in the heart. Induced GDF15 expression by adenoviral-mediated gene transfer decreased agonist-induced hypertrophy of neonatal cardiomyocyte cultures through activation of SMAD2/3. Moreover, *GDF15* knockout mice presented enhanced cardiac hypertrophy in response to pressure overload stimulation (Xu et al., 2006). However, the *GDF15* gene was found induced in microarray analyses of neonatal rat ventricular cardiomyocytes submitted to biomechanical stress as a model of pressure overload (Frank et al., 2008).

**FIGURE 1:**
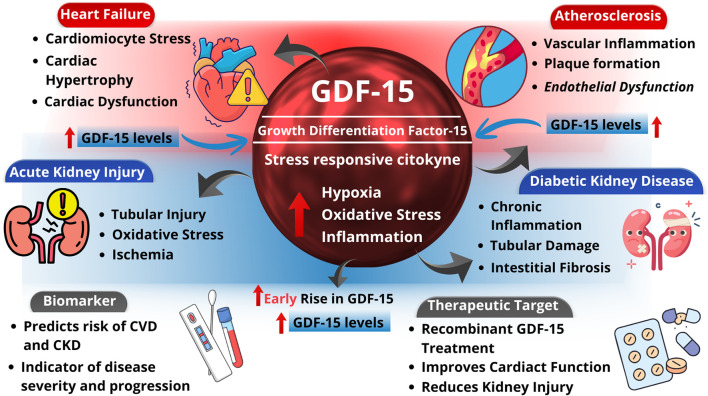
Role of GDF-15 as an emerging biomarker in cardiovascular and kidney disease. Under physiological conditions, growth differentiation factor-15 (GDF-15) is minimally expressed in cardiovascular and renal tissues. In response to pathological stressors such as inflammation, oxidative stress, ischemia, and tissue injury, *GDF15* expression is upregulated in cardiomyocytes, endothelial cells, and renal cells. Elevated circulating GDF-15 levels are associated with cardiovascular disease (CVD) and kidney dysfunction, reflecting disease severity and prognosis. GDF-15 exerts cardioprotective and renoprotective effects by modulating inflammatory pathways, cellular stress responses, apoptosis, and metabolic adaptation, highlighting its potential utility as both a biomarker and therapeutic target.

Experimental studies in rodents have shown that GDF-15 levels increased in serum and cardiac tissue following myocardial infarction (Kempf et al., 2011; Wei et al., 2023). *GDF15* knockout mice subjected to myocardial infarction exhibited enhanced inflammatory cell infiltration, cardiac rupture, fibrosis, and mitochondrial dysfunction, suggesting that GDF-15 exerts cardioprotective effects by regulating inflammatory responses, AMPK signaling, mitochondrial function, and oxidative stress (Kempf et al., 2011; Yuan et al., 2025), as depicted in Figure 1.

Furthermore, GDF-15 treatment decreased body weight, food intake, metabolic parameters, and heart weight and improved parameters of left ventricular function in an experimental model of cardiometabolic syndrome with established heart failure and preserved ejection fraction in rats (Stolina et al., 2020). *GDF15* silencing by tail vein injection of lentivirus in a rat model of heart failure with preserved ejection fraction preserved cardiac function, inhibited myocardial fibrosis, and reduced markers of oxidative stress, inflammation, and apoptosis (Meng et al., 2025). Cardiac *GDF15* mRNA expression and plasma levels were found to be increased in a mouse model of heart failure associated with cachexia. Mouse treatment with a monoclonal antibody against mouse GDF-15 prevented cachexia and slowed the worsening of cardiac function. In patients with heart failure, GDF15 plasma levels were also associated with cachexia (Takaoka et al., 2024).

In summary, *GDF15* is upregulated under injury, inflammation, and metabolic stress, suggesting an adaptive function aimed at preserving tissue homeostasis. While most studies indicate cardioprotective and anti-inflammatory effects, some discrepancies highlight that further research is needed to clarify its context-dependent actions and therapeutic potential in cardiometabolic and renal diseases.

## 3 GDF-15-targeted therapy and drug response in oncology

Clinical trial findings and perspectives on GDF-15-targeted therapy in oncology were recently summarized elsewhere (Sugiyama et al., 2025). Moreover, current advances in GDF-15-targeted drug development, including fusion proteins, and the challenges in developing GDF-15-targeted therapeutics were comprehensively evaluated elsewhere (Tian et al., 2025). For example, the GFRAL-Fc fusion protein showed therapeutic benefits through metabolic regulation and immune remodeling, and validated GDF-15 targeting as a viable strategy to overcome programmed death-1 inhibitor resistance in hepatocellular carcinoma, but these findings warrant further clinical validation (Shi et al., 2025).

Sorafenib resistance remains a major challenge to therapy for advanced hepatocellular carcinoma. Noteworthy, hypoxia-induced HIF1A was recently uncovered to promote sorafenib resistance via the NSUN2-mediated stabilization and upregulation of *GDF15*, and this novel mechanism involving the HIF1A/NSUN2/GDF15 axis offers a therapeutic target to overcome sorafenib resistance (Wang et al., 2025). Similarly, targeting the IRE1α/XBP1-GDF15 axis may represent an actionable strategy to improve chemoimmunotherapy efficacy in cervical cancer (Cao et al., 2025). Moreover, the complex role of GDF-15 on prostate cancer metabolism, chemoresistance, and metastasis were recently reviewed (Zhu et al., 2025).

Mitotane remains a mainstay of therapy for adrenocortical carcinoma, in which the response rates to immune checkpoint inhibition are disappointing. Mitotane was shown to increase GDF-15 levels and to be associated with poor response to immune checkpoint inhibition (Weigand et al., 2025). Prognostic significance of *GDF15* expression was also evaluated in non-small cell lung cancer tumor tissues, in which poorer response to nivolumab was associated with increased *GDF15* expression, suggesting that GDF-15 is a potential prognostic biomarker for immunotherapy efficacy response (Akdogan et al., 2024).

## 4 GDF-15 as a potential biomarker for preeclampsia (PE)

PE is a worldwide leading cause of maternal-fetal morbidity and mortality defined as maternal hypertension after 20 weeks of pregnancy, which may occur along with proteinuria or other indications of renal insufficiency, thrombocytopenia, liver dysfunction, pulmonary edema, and cerebral disturbances (ACOG, 2013). PE is characterized by abnormal spiral artery remodeling, placental ischemia, oxidative stress, and angiogenic imbalance, thereby resulting in widespread maternal endothelial dysfunction and end-organ damage (Phipps et al., 2019). Importantly, PE has been linked with future risk for CVD, and there is evidence for an association of PE with renal disease (Luizon et al., 2023).

Given the increased *GDF15* expression in the placenta and established links with cardiovascular dysfunction, it is also being investigated as a potential circulating biomarker for PE. Plasma GDF-15 levels increase during pregnancy (Moore et al., 2000), possibly supporting gestational maintenance, as reduced maternal serum levels in early pregnancy have been associated with spontaneous abortion (Fejzo et al., 2018). In PE, lower GDF-15 levels have been reported in later gestation (Wischhusen et al., 2020), however findings are inconsistent: while some studies found increased GDF-15 levels (Sugulle et al., 2009; Temel Yuksel et al., 2018; Cruickshank et al., 2021; Jacobsen et al., 2022), others reported decreased GDF-15 levels (Chen et al., 2016; Battarbee et al., 2024). GDF-15 has also been linked to nausea, vomiting, and hyperemesis gravidarum (Lockhart et al., 2020). Notably, PE shares several common mechanistic pathways with CVD, such as endothelial dysfunction and immune dysregulation (Countouris and Bello, 2025). Since GDF-15 is an inflammatory and stress-induced cytokine its expression is often increased upon tissue injury (Wischhusen et al., 2020), and it has been proposed as a circulating cardiovascular biomarker for PE (Cruickshank et al., 2021; Jacobsen et al., 2022). Due to the lack of effective predictive markers, further research is needed to clarify the role of GDF-15 on pathophysiology and as a potential biomarker for PE.

Managing maternal hypertension is a priority as a treatment strategy for PE (Townsend et al., 2016; Wang et al., 2023). Antihypertensive drugs have the potential to prolong gestation, decreasing obstetric and perinatal complications in PE (Berzan et al., 2014; ACOG, 2020). However, 40% of pregnant women with PE are nonresponsive to antihypertensive therapy with methyldopa, nifedipine, hydralazine, and this subgroup is more susceptible to develop adverse maternal and fetal outcomes (Luizon et al., 2017; Luizon et al., 2023). This scenario highlights the need for therapeutic options for PE management. Novel therapies targeting the pathophysiology of PE are being investigated, including metformin (Lee et al., 2025). Current evidence for the use of metformin during pregnancy in various maternal subgroups indicate that metformin might help prevent PE, but this is still unclear, and its potential role in the treatment of PE is ongoing research (McEvoy et al., 2025).

Notably, *GDF15* was found to be upregulated by metformin in primary human hepatocytes (Luizon et al., 2016). Regulation near the *GDF15* locus might be focused on further studies about the underlying epigenetic mechanisms implicated in cardiovascular and renal diseases, as previously shown (Cruz et al., 2021). Indeed, a putative regulatory region located 10 KB away from the promoter region of *GDF15* gene harbors variation found to be associated with both a GWAS lead SNP rs888663 for GDF-15 levels and a eQTL-SNP for GDF-15 levels, which were found to be located within a large region of the active histone marker activated by metformin (Linhares et al., 2020)., Functional characterization of the non-coding SNP rs888663 identified a novel enhancer within the *GDF15* locus, with the rs888663 T allele exhibiting higher enhancer activity (Pereira et al., 2026). This scenario highlights the importance of taking into consideration the drug-responsive regulatory elements (Luizon and Ahituv, 2015). Compared with healthy pregnancies, plasma GDF-15 levels were significantly lower in gestational hypertension and PE, and these patients who were carries of the TT genotype for the *GDF15* SNP rs1059369 or the ‘T,T’ haplotype formed by SNPs rs888663 and rs1059369 showed reduced GDF-15 levels (Pereira et al., 2026).

Noteworthy, the effects of GDF-15 may also depend on its concentrations, and a longitudinal study of these concentrations along pregnancy would be relevant to establish it as a pharmacological biomarker, as previously shown for visfatin/NAMPT (Ceron et al., 2023; Nunes et al., 2023). PE is characterized by low bioavailability of nitric oxide, which were correlated with potential biomarkers for PE (Pereira et al., 2021; Luizon et al., 2022; Pereira et al., 2024) linked to endothelial dysfunction and pathways underlying cardiac and vascular remodeling (Ceron et al., 2022). Therefore, the interplay among GDF-15 and these potential biomarkers for PE should be further explored.

GDF-15 links placental dysfunction, endothelial injury, and cardiovascular stress, and thereby emerges as a potential biomarker and therapeutic target in PE. Its expression is influenced by genetic variation and epigenetic factors and can be modulated by pharmacological agents such as metformin. Understanding how GDF-15 interacts with pathways involved in endothelial dysfunction and NO signaling may improve early diagnosis and guide targeted therapies. Further studies integrating longitudinal biomarker measurements and mechanistic analyses are needed to clarify its role in pregnancy-related hypertensive disorders and their associated cardiovascular complications.

## 5 Conclusion

We discussed potential mechanisms underlying the effect of GDF-15 on endothelial dysfunction and cardiovascular and renal risk, along with findings regarding GDF-15 as therapeutic target in cancer and as a potential biomarker for PE. Future studies of translational research are needed to evaluate the clinical relevance of these mechanisms and the possible beneficial effects of GDF-15-based therapies in CVD and kidney disease.
